# Home Range of the Caspian Whipsnake *Dolichophis caspius* (Gmelin, 1789) in a Threatened Peri-Urban Population

**DOI:** 10.3390/ani13030447

**Published:** 2023-01-28

**Authors:** Thabang Rainett Teffo, Krisztián Katona, Gergely Babocsay, Endre Sós, Bálint Halpern

**Affiliations:** 1 Department of Wildlife Biology and Management, Institute for Wildlife Management and Nature Conservation, Hungarian University of Agriculture and Life Sciences, Páter Károly u. 1., H-2100 Gödöllő, Hungary; 2 MME Birdlife Hungary, Költő utca 21., H-1121 Budapest, Hungary; 3 Mátra Museum of the Hungarian Natural History Museum, Kossuth Lajos utca 40., H-3200 Gyöngyös, Hungary; 4 Budapest Zoo and Botanical Garden, Állatkerti krt. 6-12., H-1146 Budapest, Hungary; 5 Department of Systematic Zoology and Ecology, Institute of Biology, Doctoral School of Biology, Eötvös Loránd University, Pázmány Péter sétány 1/C, H-1117 Budapest, Hungary; 6 ELKH-ELTE-MTM Integrative Ecology Research Group, Pázmány Péter sétány 1/C, H-1117 Budapest, Hungary

**Keywords:** daily displacement, hibernation, human disturbance, radiotelemetry, reptile, snake conservation

## Abstract

**Simple Summary:**

We investigated movements of the Caspian whipsnake (*Dolichophis caspius*) in a peri-urban habitat in Budapest, Hungary. Radiotelemetry results showed that the five tracked individuals used a larger part of habitats (40.15 ha) during warmer seasons, but a relatively smaller area (1.75 ha) of a rocky mount with a high hibernaculum density during winter periods. During the main activity season, the individual home range sizes varied between 6.1 and 15.5 ha; meanwhile, the average daily displacement of the snakes was found to be between 12.6 and 36.6 m. For this study, we can conclude that Caspian whipsnakes have enough space to move around the area for foraging, but hibernating places are limited. Human activities and anthropogenic disturbances, especially around hibernacula, may exert pressure on this peri-urban snake population.

**Abstract:**

Semi-natural environments within cities can provide habitats for vulnerable reptile species. Better understanding of their habitat use and home range sizes is important for their conservation. We investigated the spatial ecology of Caspian whipsnakes (*Dolicophis caspius*) in a peri-urban habitat in Budapest, Hungary. We used radiotelemetry to track five adult snakes and analyzed their microhabitat preferences, home range sizes and daily movements. The Caspian whipsnakes intensively utilized areas covered with woody vegetation, with a high density of hibernacula. The tracked snakes used an area of 40.15 ha during the activity period from spring to autumn, but for the winter, they withdrew to a central area of 1.75 ha, abundant in hibernacula. During the activity period the individual home range sizes varied between 6.1 and 15.5 ha, estimated using the minimum convex polygon (MCP); however, for the entire datasets of the individuals, the adaptive kernel method gave the highest mean (13.8 ha), while the LoCoH-R yielded the smallest home ranges (5.19 ha). We found that the average daily displacement for the different individuals ranged between 12.6 and 36.6 m during their main activity season. In the study area, the whipsnakes currently have enough space for foraging, but the restricted spatial distribution of hibernacula, which is mainly available in the central dry rocky forest and partly in the shrubby areas, can limit the extent of the suitable habitat. Human activities and anthropogenic disturbances, especially around hibernacula, may exert pressure on this peri-urban snake population.

## 1. Introduction

Research on spatial ecology of reptiles has shown either limited space use [1,2] or reduced movements [3,4] in fragmented and urban landscapes. Reptile populations are declining due to threatening factors which include urbanization [5], conversion of natural habitats into agricultural lands, coupled with overgrazing by livestock and intensive agricultural practices [6,7,8,9,10]. Some species persist in smaller and increasingly fragmented geographic ranges [11,12], wherein the most vulnerable species require active conservation measures to ensure their survival [13]. For example, Australian tiger snakes (*Notechis scuatus*) and African rock pythons (*Python sebae*) endure limited space use and movements due to habitat fragmentation [14]. Disturbances associated with urban areas such as fragmentation, invasive species, road accidents and isolation of refugia may result in high mortality of sensitive species [11,13]. 

There is insufficient knowledge concerning the spatial behavior of species living in human-dominated landscapes [15,16,17]. In general, snakes appear to have smaller areas of utilization in disturbed and fragmented habitats (including urban landscapes). For example, the colubrid Coachwhip (*Masticophis flagellum*) and Eastern indigo snakes (*Drymarchon couperi*) maintain smaller home ranges there than in natural habitats [1,18]. However, in undisturbed habitats, large-bodied snakes maintain larger home ranges, as was observed in Northern Pinesnakes (*Pituophis m. melanoleucus*) in New Jersey pine barrens, where large males covered over 258 ha [19]. Similarly, in a nature reserve in Southern Florida, the annual home range of the Eastern indigo snake was 110 ha for females and 207–233 ha for males [20]. 

Besides habitat quality, variation of movements in snakes is also influenced by the combination of sex, season, and latitude [21,22]. For Eastern Indigo snakes in temperate zones, males establish larger home ranges and move longer distances than females [23,24]. Jellen et al. [25] demonstrated that the crotalid Eastern Massasuga (*Sistrurus catenatus*) males followed and mated with several females during the mating season. As a result, males moved larger average daily distances (21.8 m) during the mating season than outside of it (13.3 m). In general, reptiles in the temperate zones are most active during the warmer months, and are inactive during winter and seldom move within their hibernacula [26]. 

The Caspian whipsnake (*Dolicophis caspius*) is a large-bodied colubrid often exceeding two meters in total length. The distribution area of this species covers south-eastern Europe, including the Balkans and the steppe region extending eastward between the Black and the Caspian Seas [27,28]. The north-western edge of its distribution reaches North-Central Hungary [27,29]. This species inhabits dry steppe and sub-Mediterranean habitats [30,31]. In Hungary, it exists in small, isolated populations [29]: i.e., in southern Hungary (Szársomlyó Hill), along the western bank of the Danube River and on dolomite, limestone and sandstone outcrops in and around Budapest. In the Carpathian Basin, the populations have become increasingly fragmented, owing to habitat alteration [32]. The Caspian whipsnake is strictly protected in Hungary; threats to this species are not thoroughly studied, but habitat loss [30], road traffic (https://herpterkep.mme.hu/ accessed on 1 December 2022), and invasive plants [33] are considered as primary threats to its survival. 

Movement ecology of the Caspian whipsnake has not been studied yet, neither in natural nor in urban landscapes. Our study is the first to investigate the home ranges and daily movements in this species from a population inhabiting a peri-urban habitat within Budapest, the capital of Hungary. We selected an isolated population of Caspian whipsnake, inhabiting a diverse area of hilly dry rocky mount with woody vegetation surrounded by shrubby meadows and grasslands, and enclosed by closed forest stands and residential areas.

Given that Caspian whipsnakes may frequently utilize habitat patches consisting of hideouts and hibernacula, we expected a relatively high concentration of telemetry points of snakes on the dry rocky mount with woody vegetation, especially during the hibernation periods. Based on previous visual observations, no extended movements to other habitat patches were presumed. Therefore, we focused primarily on microhabitat preferences, seasonal home range sizes and overlaps and daily displacements of Caspian whipsnakes. We expected high variabilities coming from methodology of home range estimations of individual snakes, considering the sample size. Furthermore, variations in their sexual differences were also considered.

## 2. Materials and Methods

### 2.1. The Study Area

**Figure 1:**
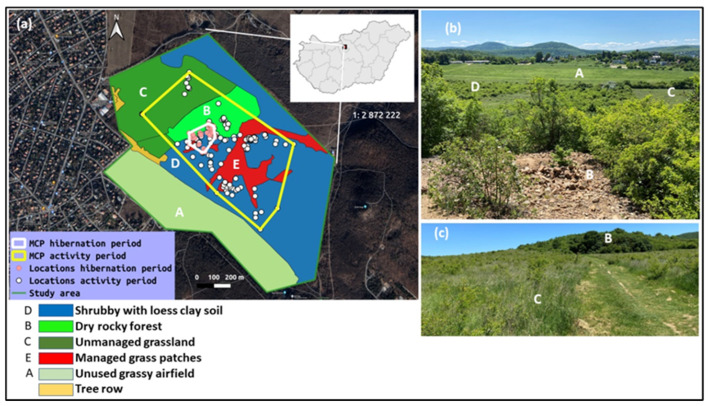
(**a**) Overall distribution of all telemetry points during activity period (*n* = 226) and hibernation period (*n* = 83) of five Caspian whipsnakes (*Dolichophis caspius*), distributed in various habitat patches estimated using the minimum convex polygon (MCP) method. (**b**,**c**) images from the study area depicting different habitat patches. Capital letters on (**a**) correspond to those on (**b**,**c**) showing some of the habitat types.

The study was conducted in a semi-natural habitat complex covering an area of 125 ha surrounded by suburban and forest environments in Pesthidegkút, Budapest, Hungary (Figure 1). Part of the study area is included in the Natura 2000 network and forms a part of the landscape protection area of the Buda Hills within Duna-Ipoly National Park. The habitat is a mosaic of grassy, shrubby, rocky patches and forest. In earlier decades, the area was heavily grazed by sheep. 

The center of the area is a rocky elevation, consisting of shallow quarries and thick sandstone piles deposited during earlier mining activities. The shrubby and open grassy areas are characterized by a combination of loess solonetz soil and alluvial meadow soils (i.e., see Pásztor et al. [34]). Recently, shrubby and woody vegetation has been replacing the grassy vegetation, creating suitable habitats for wild boar (*Sus scrofa*) and carnivores (e.g., red fox *Vulpes vulpes*). The area also serves as a recreational area for residents, which exerts heavy human disturbance (i.e., dog walking, hiking, mountain biking) on the snake population. In 2018, on the central rocky mount, an educational trail, named after Dr. Jane Goodall, was created to raise awareness of the Caspian whipsnake, and other protected wildlife of the area, to the public. 

### 2.2. Handling the Snakes and Transmitter Implantation

Radio-tracking of the Caspian whipsnakes has been carried out along with a long-term monitoring of the population by the Amphibian and Reptile Conservation Group of MME BirdLife, Hungary. The snakes were caught by hand. Altogether, 79 specimens have been caught, and out of these, 68 were identified as separate individuals. For each captured snake we measured mass (to the nearest gram), snout-to-vent length, and tail length (to the nearest 1 mm). We also photographed the head, ventral side and tail, with intention of counting scalation later for individual identification, and also recorded sex (by assessing tail shape and relative tail length). Between 2016 and 2019, radio-transmitters were implanted into two males and three females. Immediately after capturing, the snakes were transferred to the veterinary clinic of the Budapest Zoo. They were anaesthetized with injectables (ketamine and midazolam) and inhalant anesthetics (Sevoflurane) to implant VHF radio-transmitters (manufactured by the Wildlife Ecology Institute, Vienna, Austria) surgically into the coelomic cavity. The ratio of transmitter mass (core body: 17 × 10 × 7 mm, weight = 2.5 g with a flexible antenna of 8 cm) to snake mass was below the 5% threshold value, as is customary in radiotelemetry of snakes [35]. All surgeries were carried out within twelve hours after capture and all snakes were released after 24 h of observation care. The lifespan of the transmitters was indicated as a minimum of one year, but they operated for slightly longer (less than 18 months). For two individuals, (Vali and Lili) we replaced the transmitters once. 

The low sample size Imposed some limitations on the interpretation of our results, but because of the vulnerability of the population, we had to limit the number of specimens supplied with surgically implanted transmitters. For a public conservation campaign, the tracked snakes were named (Table 1); two of them received names honoring Dr. Valery Jane Goodall, who had taken a special interest in the conservation project of this population. 

### 2.3. Radiotelemetry

We tracked the snakes with a Televilt RX900 (Followit AB, Lindesberg, Sweden) receiver with YAGI-antenna. The snakes were localized at least once a week, but occasionally twice. The localization frequency during winter was half of that in summer months. We followed each of them at least for a year, but the two of them with replaced transmitters were tracked for two and three years, respectively. When a snake was located, we recorded the following data: the coordinates (using a Garmin Map64x handheld GPS with Universal Transverse Mercator [UTM] WGS 84 projection, working with 0–10 m precision), the date and time, whether the snake was visible above ground or hid underground and the vegetation type in which the snake was found. We transformed the coordinate reference system (CRS) from WGS-84 to the uniform national projection system for the Hungarian civilian base maps (HD/72 EOV).

### 2.4. Data Analysis

#### 2.4.1. Habitat Availability and Use 

We distinguished five microhabitat categories within the study area, as shown in Figure 1: (A) recently abandoned grass-covered airfield (regularly mowed), (B) dry rocky open forest (mostly a secondary habitat of a former quarry, with large heaps of mined stones providing hibernacula), (C) unmanaged grassland (where shrub encroachment is not actively mitigated by humans), (D) shrublands neighboring the rocky areas and (E) managed grassy patches (where shrubs have been occasionally removed). We digitized the borders of each area by creating polygons on a Google satellite map using QGIS software, Madeira, Spain (v3.4.11). We used the vector analysis tool to count the number of snake locations in each habitat type (i.e., habitat use).

#### 2.4.2. Calculating Home Range Size

Previous studies showed that various home range estimation methods give highly variable results [36]. Therefore, to estimate home ranges, we included four traditionally used common methods: minimum convex polygon (MCP), fixed kernel density estimation (KDE) and adaptive KDE in 90% utilization distributions [37,38], (UD) and local convex hull (LoCoH-R) [39] using QGIS software (v3.4.11) and the OpenJUMP HoRAE 1.15 toolbox [40]. We had no intention of making a thorough methodological comparative investigation. Instead, we aimed to better describe the home range size of the species, recognizing the variability coming from the different estimating approaches. We estimated home range sizes for the entire year and separately for the activity period (see definition below). Overlaps of home ranges during the active season were calculated using the MCP method. To achieve this, we used the geoprocessing tool in QGIS to calculate the area (in ha) and the percentage of overlap between MCPs of individual snakes. Overlap was primarily given as the ratio of overlapping area to the total area covered by the home ranges of the pairs. We also calculated the proportion of the overlapping area within the home range of each individual of the pairs.

#### 2.4.3. Daily Movements

We calculated the linear distance in meters between consecutive telemetry locations using the general distance formula in Microsoft Excel 2010, where X1; Y1 and X2; Y2 coordinates represented the two consecutive recorded points. Points which were found within 10 m of distance from each other were merged into one geometrically calculated midpoint location to eliminate the confounding effect of GPS location inaccuracy. Subsequently, the average daily displacement of each snake was calculated by dividing the linear distance between the consecutive locations by the number of days passed between the two recordings. We are aware that it may be a gross underestimation of the total distance travelled during these intervals (daily movement distance, as the sum of straight-line distances between consecutive locations [41]). 

We divided the yearly activity of the snakes to an activity and a hibernation period. Since Caspian whipsnakes in Hungary hibernate during winter, we focused on the activity period in our analyses. For each snake, the activity period was defined individually from the beginning of the post-hibernation period when, at least three consecutive times, the snake was observed above-ground and all three of its movements were longer than 10 m. By our definition, the hibernation period started when, at the end of the activity period, at three consecutive times, the snake was found underground and each time, the distances were found to be less than 10 m. The lengths of these periods varied slightly among the snakes; however, our data showed that the activity period lasted roughly from May to August, and the hibernation period from September to either April or May. However, based on our direct field observations snakes may be active from late March to late November.

### 2.5. Statistical Analyses

Jacobs’ selectivity index was calculated and the chi^2^-test with Bonferroni correction was conducted to determine the relative availability of different microhabitats and their preference [42] by snakes. The analysis was performed separately all year round on individual datasets and on the combined dataset (all telemetry locations of five snakes combined). All other datasets were tested for normality using the Shapiro-Wilk test. The average yearly home range sizes of the snakes, as calculated using four estimation methods, and average daily displacements of consecutive months were compared using a repeated measures ANOVA, followed by a Tukey-Kramer post-hoc test and a Friedman ANOVA with Durbin-Conover pairwise comparisons, respectively. To compare the daily displacements among the individuals, the Kruskal-Wallis test, followed by Dwass-Steel-Critchlow-Flinger (DSCF) pairwise comparisons, was used. However, due to the small sample size, we could not compare the home range sizes of individuals or sexes for the activity period. We evaluated the relationship between body weight of the tracked snakes and their home range size, estimated using minimum convex polygon during the activity period, and calculated this using the Spearman correlation test. Similarly, we evaluated the association between body weight and average daily displacements. All statistical analyses were performed using Jamovi v1.1.9.0 [43].

## 3. Results

We obtained 309 location points from the five individuals (Table 1).

During the entire study period, the tracked individuals of this Caspian whipsnake population used an area of only 40.15 ha. The snakes aggregated within a small area (1.75 ha) in the central rocky patch during hibernation, but their movements in the activity period covered a much larger area with various habitat characteristics (Figure 1).

**Figure 2:**
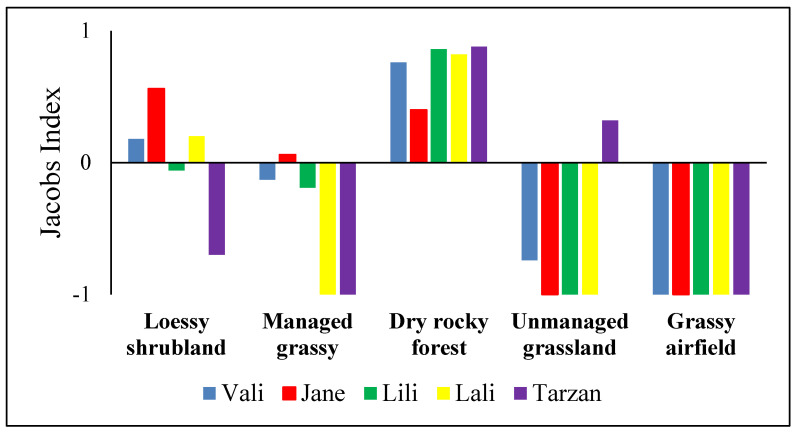
Jacobs’ index values showing microhabitat preferences of Caspian whipsnake (*Dolichophis caspius*) individuals in the study area.

By combining all telemetry points into one dataset, we found that Caspian whipsnakes preferred the dry rocky forested patch with a high density of hibernacula and dens, and the neighboring shrubland areas (Jacobs’ index: D = 0.78 and 0.10, respectively) but avoided the managed grassy patches and the unmanaged grassland (D = −0.40 and −0.38, respectively). The nearby grassy airfield was entirely avoided (D = −1) by the snakes. Considering individual preferences, (Figure 2) snakes showed similar patterns, except that Tarzan was never tracked in the shrubland but preferred the unmanaged grassland, while Jane was the only snake preferring the managed grassy patches.

**Figure 3:**
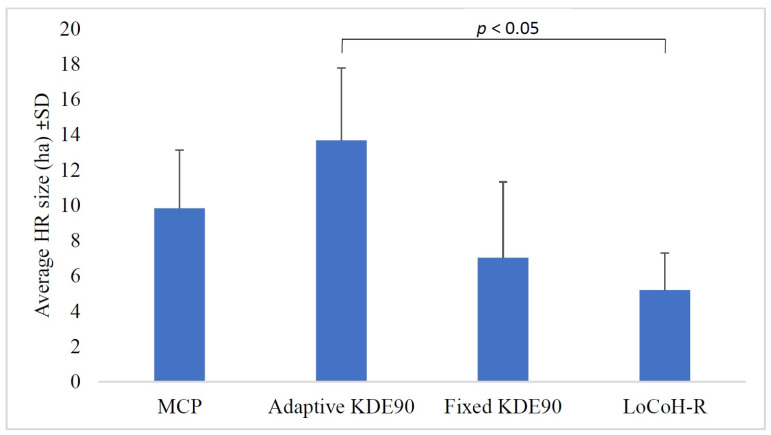
Average home range (HR) sizes calculated using the entire tracking dataset of all the years of tagged Caspian whipsnakes (*Dolichophis caspius*) in the study area estimated using four different methods. There was a significant difference between adaptive KDE90 and LoCoH-R (*p* < 0.05).

The estimated average individual home range sizes calculated using all the (year-round) tracking data of each snake (2016–2019) ranged between 5.19 ha (LoCoH-R) and 13.8 ha (adaptive KDE90). We revealed a significant difference between the mean home range sizes calculated with the four different methods used (repeated measures ANOVA: F = 5.342; df = 3; *p* = 0.014). There was a significant difference between adaptive KDE90 and LoCoH-R (Tukey Kramer post-hoc test: *p* < 0.05) and no significant difference was found among the estimates of other methods (*p* > 0.05) (Figure 3).

**Figure 4:**
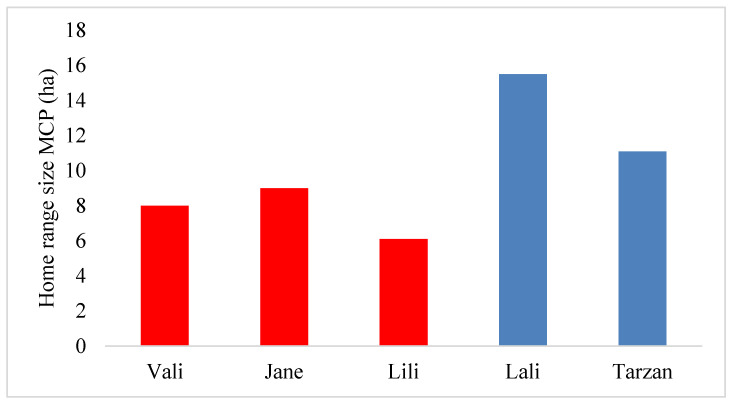
Individual home range sizes of Caspian whipsnakes (*Dolichophis caspius*) calculated using MCP during the activity period. (Red bars: females; blue bars: males).

During the activity periods, individual snakes established home ranges of a size between 6.1 and 15.5 ha, calculated using MCP. In this active interval, home ranges of the two males were larger than those of the three females (Figure 4). There was a weak positive correlation between body weight and home range size, calculated using MCP (Spearman correlation test: r = 0.25; *p* = 0.002). During the hibernation periods the individual home ranges were reduced to less than 0.3 ha in the central dry rocky patch, with woody vegetation consisting of hibernacula.

**Figure 5:**
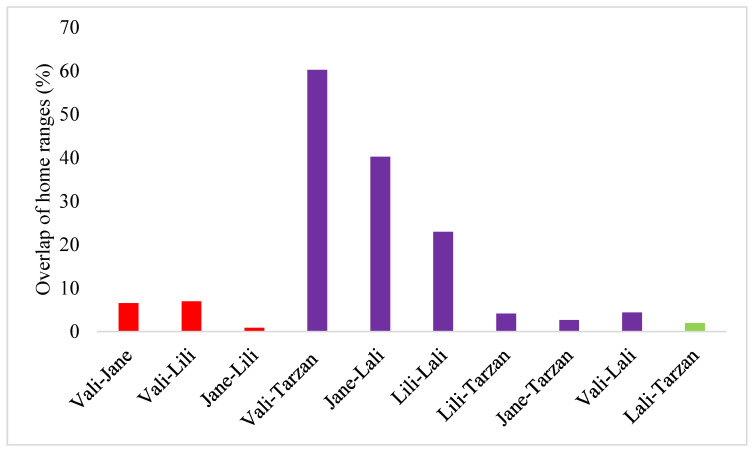
Overlap of the individual home ranges of Caspian whipsnakes (*Dolichophis caspius*) as a percentage of the total area covered by the home ranges of the pairs (%), calculated using MCP during the activity period. (Red—female-female overlap, purple—female-male overlap and green—male-male overlap).

In most cases (7/10) the overlaps of individual home ranges were relatively small (<10%) during the activity period, however, for some female-male pairs the overlap reached as high as 23, 40 and 60% in three pairings (Figure 5). When the overlaps were expressed as the percentage of the home range of each pair, the overlaps averaged 24% in male and 43% in females in female-male pairs.

**Figure 6:**
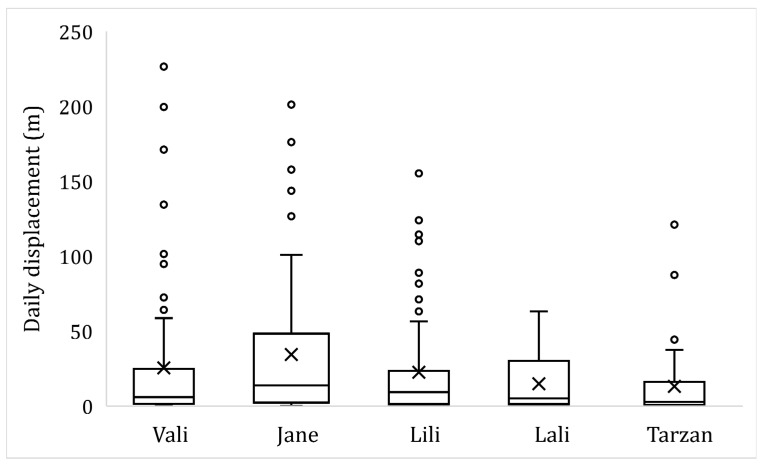
Daily displacements of five Caspian whipsnakes (*Dolichophis caspius*) during the activity period (box lines show medians and upper and lower quartiles, whiskers provide the minimum and maximum values, circles are the outlier values, and X-s give the means).

There was no significant difference among the individual daily displacements of the five snakes during the activity period (Kruskal-Wallis test: X^2^ = 9.23; df = 4; *p* = 0.056). A female snake (Jane) showed the largest, meanwhile a male (Tarzan) showed the smallest displacements (Figure 6). There was no association between the body weight and average daily displacement of the snakes (Spearman correlation: r = −0.32; *p* = 0.08). The maximum absolute value of the daily displacement during the activity period was found to be 226 m by a female (Vali).

**Figure 7:**
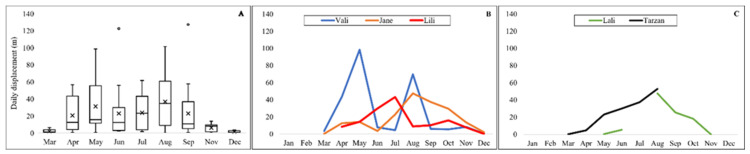
Daily displacement of five Caspian whipsnakes (*Dolichophis caspius*), throughout the years. (**A**–**C**): Average daily displacement in females and males respectively, throughout the years.

However, we found a significant temporal variation in the daily displacements of snakes throughout the year (Friedman ANOVA: X^2^ = 27; df = 9; *p* = 0.001). Durbin-Conover pairwise comparisons showed that the daily displacements of the snakes in March and December were significantly lower than those of the other months. In August, the movements were longer than in June or July or in the autumn months (Figure 7A). Except for a peak in August, no discernible temporal pattern of daily displacement distance was identified for either sex (Figure 7B,C).

## 4. Discussion

We present the first results for the spatial ecology of the Caspian whipsnake in a peri-urban environment. The five radio-tracked Caspian whipsnakes together used 40.15 ha, but within this, the individual home ranges averaged only 5.19 to 13.8 ha, depending on the home range estimation methods used. These findings reflect relatively small home range sizes, similar to other temperate zone colubrids such as the smooth snake (*Coronella austriaca*) living in disturbed landscapes, maintaining home range of less than 4 ha [44]. Although studies on movement ecology of the Caspian whipsnake have been unavailable so far, we compared this data to findings on similar large-bodied snakes in natural landscapes, for example, the Coachwhip (*Masticophis flagellum)*, which tends to establish larger home ranges (~150 ha) [1]. For Caspian whipsnakes, the smaller home ranges may be due to the aggregation of shelters in the central rocky mount. Bauder et al. [45] found that habitat heterogeneity and low urban intensity resulted in reduced resource dispersion (prey, refuge) leading to smaller home ranges in threatened Eastern Indigo snakes. Similar findings are observed in our study, as the Caspian whipsnake lives in a mosaic habitat in a peri-urban area. 

Our results showed that home range sizes were influenced by the method of calculation we used. Earlier research stated that home range estimations using MCP may be more accurate than adaptive and fixed kernel when the sample size is small; in that case, adaptive kernel methods tend to overestimate home ranges more frequently [36]. However, the fixed kernel method has shown the best performance in simulation trials of home range estimators [46]. The LoCoH method underestimates home ranges by identifying “hard boundaries” (sharply separated spots, such as steep slopes, mountains, roads, rivers, etc.), and excluding them from calculation [47]. At the same time, the method can precisely outline the spatial movements of individuals for which the absence of records indicates real gaps in occurrence. Revealed variability due to the different types of estimates support these trends, as we observe similar a trend in our findings in the case of adaptive kernel and LoCoH-R.

In our study, during the activity periods, snakes were localized both above and underground; meanwhile, during hibernation periods, the snakes were found invariably underground, but it is possible that they were moving under or above ground between two consecutive winter locations. Because of the latter, this kind of separation of the active and hibernation periods we performed is somehow arbitrary, potentially leading to the truncation of the active period. However, we could decide whether an animal is hibernating or not, based only on the lack of direct visual observations of the emerged individual above ground and the highly reduced length of their daily displacements. During active periods snakes in temperate zones establish larger home ranges, owing to hunting and searching for mating partners (e.g., [21]). We found that the snakes showed an average daily displacement of a range 12.6 m to 36.6 m, with the longest distance being 226 m covered by Vali during the activity period. However, we are aware that with the localization frequency we used, we missed movements between two localization events (i.e., the snakes could make repeated long-distance movements between two locations); therefore these averages could be underestimated. During our fieldwork, we observed individuals moving distances exceeding the above averages within a few hours.

According to Frank and Dudás [48], Caspian whipsnake mating peaks in spring (April and May). Although we did not investigate this, in spring, gravid females are more likely to have smaller home ranges than males; hence, we could observe short movements by some females. We found that males had larger home ranges during the activity period as compared to females as they travel widely while searching for mating partners [49]. Similarly, in Eastern Indigo snakes, males had larger home ranges with a significant increase during the breeding season [18,24]. Reading [44] reported similar findings on smooth snakes in England. The increased spatial use of male Caspian whipsnakes may be due to males actively searching for females as reported on Eastern indigo snakes [18]. However, the longest revealed displacement was related to a female (Vali), which may be related to her higher foraging activity after parturition to cover higher reproductive costs [50].

In our study, Caspian whipsnakes entirely avoided some patch types: the grassy airfield and the residential area. However, they preferred other patches such as the dry rocky outcrops within the woody vegetation for hibernating in the available hibernacula, and shrubby areas, including grassy openings for foraging, all of which forms a typical habitat structure that Caspian whipsnakes prefer [30,51]. Similarly, Reading and Jofré [52] reported that Grass snakes in Northern England avoided open grazing lands and strongly preferred habitat boundaries and interfaces. Moreover, we tracked one snake (Tarzan) several times on the unmanaged grassland, which was avoided by the others. The open surfaces on the rocks and grassy openings in the shrubland can provide fast warming basking sites. Additionally, the grass fields on the loess provide substrate for burrowing to the European ground squirrel (*Spermophilus citellus*) and large colonies of the common vole (*Microtus arvalis*), both providing ample biomass of prey for the large-bodied whipsnakes. On the other hand, the still regularly mowed airfield and the human residential area may be perceived by the snakes as unsuitable or risky with high chances of mortality by mowing machines and direct killing by humans or dogs. During hibernation, the snakes were confined in the smaller central rocky area with woody vegetation. We suggest that, for this species, the woody vegetation was not the priority. It is probable that, for this large-bodied snake, only the part of the habitat ridden with deep-reaching cracks and stone piles left by the former quarry provided suitable hibernacula where they find thermally optimal cavities, even in harsh cold spells of the winter. Similar findings were reported on communal hibernaculum use of seven snake species, including Caspian whipsnakes, in Bulgaria [53]. 

In that research study [53], Caspian whipsnakes used the same microhabitats around the hibernacula during the rest of the active period after spring dispersal (pre-hibernation), not moving very far from it. In our research, we found that during the activity period the areas within which the snakes used underground burrowing places were partly shifted from the rocky area to the neighboring shrubby and grassy areas. This suggests that, in summer, they use burrows of rodents as temporal hiding places and they stay away from their permanent dens for longer periods. We have direct observation of Jane, Tarzan and Lili using these burrows; Jane also spent a whole winter in a rodent burrow (not in the cavities of the quarries) in the central hibernation area. Many snakes in temperate zones show syntopic hibernation [53]. We could observe Vali and Lili sharing the same burrow over consecutive winters. Moreover, Tarzan used the burrow for wintering that Vali was using before. However, the species may aggregate for sheltering due to limited hibernation areas and not because they want to share the hibernacula. For the Caspian whipsnakes, the best hiding places could be in the central rocky area (1.75 ha); they cohabit in this patch during hibernation. These results suggest that areas used by snakes for burrowing can be seasonally limited, since, for hibernation all individuals tend to find shelter in the rocky patches in natural rock fissures, and do not utilize the holes of ground squirrels or other rodents in the grassy areas during winter (except in the abovementioned case of Jane). However, other studies found that small home ranges occurring in altered landscapes may be associated with high quality of habitat [52].

Our results show that, during the activity period, overlaps of individual home ranges were small (<10%); however, for half of the female-male pairs, the overlap extended this level and could reach 60%. There were small overlaps, especially between snakes of the same sex, suggesting that Caspian whipsnakes may actively avoid conspecifics of the same sex while foraging in the area. Similar findings were reported when investigating spatial overlap of Eastern Indigo snakes [54], as it was found that male home ranges often or completely overlap female home ranges, whereas in snakes of the same sex were much less overlapping. However, considering that large number of individuals were identified (*n* = 68) in the area the interpretation of these findings call for caution.

## 5. Conclusions

The tagged Caspian whipsnakes were found in a limited area (40.15 ha) relative to the size of the study area potentially occupiable by snakes (125 ha). The individual home ranges were much smaller than the entire available habitat, no matter which estimation method was used. Males tend to establish larger home ranges, but they had somewhat shorter displacements per day than females during the activity period. However, to understand these variations further research should consider spatial patterns for gravid and nongravid females during mating and non-mating seasons. 

Our results suggest that in this area the snakes utilize variety of patches for different reasons: in winter, they aggregate in a smaller patch (the central dry rocky mount covered with woody vegetation) primarily due to high availability of hibernacula, whereas during the activity period, shrubby vegetation and some open grassy patches are also used for foraging.

Based on our results, we conclude that the population size of the Caspian whipsnake in our study area will not expand unless new similar rocky outcrops are freed by thinning the dense forests at nearby areas. Small, protected patches can only support a fewer number of individuals. Therefore, to maintain and increase the population of Caspian whipsnakes in this peri-urban area, conservation efforts should be channeled towards habitat management interventions such as clearing dense woody patches on the surrounding dolomite hillsides and opening up grass fields by partially removing shrubby thickets.

## Figures and Tables

**Table 1:** Name, sex (F: female or M: male), body weight (g), total length (cm), length of the tracking period (first and last day), number and seasonal distribution of above-ground and underground locations of radio-tracked Caspian whipsnakes (*Dolichophis caspius*).

Name	Sex (F/M)	Body Weight (g)	Total Length (cm)	Date of First Localization	Date of Last Localization	Number of Localization Points				
						Total	Above Ground	Under- Ground	Activity Period	Hibernation Period
Vali	F	464	148	22 May/2016	19 Jun/2019	92	37	55	62	30
Jane	F	266	120	22 May/2016	07 Jul/2017	68	29	39	60	8
Lili	F	398	143	02 Aug/2016	19 Oct/2018	78	42	36	64	14
Lali	M	778	159	01 Jun/2018	09 Aug/2019	27	12	15	18	9
Tarzan	M	1098	185	26 Aug/2016	06 Jul/2017	44	6	38	22	22

## Data Availability

Data for this research is available from the authors upon request.
